# The cancer secretome: a reservoir of biomarkers

**DOI:** 10.1186/1479-5876-6-52

**Published:** 2008-09-17

**Authors:** Hua Xue, Bingjian Lu, Maode Lai

**Affiliations:** 1 Department of Pathology, School of Medicine, Zhejiang University, PR China; 2 Department of Surgical & Cellular Pathology, the Affiliated Women's Hospital, School of Medicine, Zhejiang University, PR China

## Abstract

Biomarkers are pivotal for cancer detection, diagnosis, prognosis and therapeutic monitoring. However, currently available cancer biomarkers have the disadvantage of lacking specificity and/or sensitivity. Developing effective cancer biomarkers becomes a pressing and permanent need. The cancer secretome, the totality of proteins released by cancer cells or tissues, provides useful tools for the discovery of novel biomarkers. The focus of this article is to review the recent advances in cancer secretome analysis. We aim to elaborate the approaches currently employed for cancer secretome studies, as well as its applications in the identification of biomarkers and the clarification of carcinogenesis mechanisms. Challenges encountered in this newly emerging field, including sample preparation, in vivo secretome analysis and biomarker validation, are also discussed. Further improvements on strategies and technologies will continue to drive forward cancer secretome research and enable development of a wealth of clinically valuable cancer biomarkers.

## Introduction

Cancer remains the major devastating disease throughout the world. It is estimated that cancers are responsible for over 6 million lives per year worldwide with an annual 10 million or more new cases. In developing countries, cancers are the second most common cause of death, which comprise 23–25% of total mortality. Despite advances in diagnostic imaging technologies, surgical management, and therapeutic modalities, the long-term survival is poor in most cancers. For example, the five-year survival rate is only 14% in lung cancer and 4% in pancreatic cancer [1,2]. Obviously, the frustrating therapeutic effects in cancer lie in the fact that the majority of cancers are detected in their advanced stages and some have distant metastases, rendering the current treatment ineffective. It is widely accepted that early diagnosis and intervention are the best way to cure cancer patients [3,4]. Cancer biomarkers provide diagnostic, prognostic and therapeutic information about a particular cancer and show their ever-increasing importance in early detection and diagnosis of cancer [5-8].

Over the past several decades, enormous efforts have been made to screen and characterize useful cancer biomarkers. Some important molecules including carcinoembryonic antigen (CEA), prostate specific antigen (PSA), alpha-fetoprotein (AFP), CA 125, CA 15-3 and CA 19-9, have been identified. They are commonly employed in clinical diagnosis. Unfortunately, most biomarkers are not satisfactory because of their limited specificity and/or sensitivity [9,10]. Therefore, there is an urgent need to discover better potential biomarkers in clinical practice.

Currently, we are in an era of molecular biology and bioinformatics. Many novel approaches have been introduced to identify markers associated with cancer. Proteomic profiling is one of the most commonly applied strategies for cancer biomarker discovery. There are two general differential proteomic strategies: comparing protein patterns in cancer tissue with their normal counterparts, and comparing plasma/serum from cancer patients with those from normal controls. As suggested by Liotta [11]: "the blood contains a treasure trove of previously unstudied biomarkers that could reflect the ongoing physiologic state of all tissues", and the latter, therefore, appears to be more attractive. However, the prospects of blood proteomics are challenged by the fact that blood is a very complex body fluid, comprising an enormous diversity of proteins and protein isoforms with a large dynamic range of at least 9–10 orders of magnitude [12]. The abundant blood proteins, such as albumin immunoglobulin, fibrinogen, transferrin, haptoglobin and lipoproteins, may mask the less abundant proteins, which are usually potential markers [13]. Several procedures have been made to remove these more abundant proteins before proteomic analysis: for instance, the Cibacron blue dye method is used for removing albumin, Protein G resins or columns for IgG, and immunoaffinity for several abundant proteins including IgG and albumin [14-18]. Nevertheless, these methods may sacrifice other proteins by nonspecific binding, thus lowering the screen efficiency [19].

Given the above-mentioned major limitations in blood proteomics, scientists are seeking other methods for cancer biomarker discovery. The term "secretome" was first proposed by Tjalsma et al. [20] in a genome-based global survey on secreted proteins of Bacillus subtilis. In a broader sense, the secretome harbors proteins released by a cell, tissue or organism through classical and nonclassical secretion [21]. These secreted proteins may be growth factors, extracellular matrix-degrading proteinases, cell motility factors and immunoregulatory cytokines or other bioactive molecules. They are essential in the processes of differentiation, invasion, metastasis and angiogenesis of cancers by regulating cell-to-cell and cell-to-extracellular matrix interactions. More importantly, these cancer secreted proteins always enter body fluids such as blood or urine and can be measured by non-invasive assays. Thus, cancer secretome analysis is a promising tool supporting the identification of cancer biomarkers. The current review will focus on the technical aspects, applications and challenges in cancer secretome research.

## Approaches for cancer secretome analysis

In recent years, the emerging technologies in life science, especially that of proteomic research, have greatly accelerated studies on the cancer secretome. Generally, these methods can be categorized into two groups, namely genome-based computational prediction and proteomic approaches.

### The genome-based computational prediction

These approaches are characterized by a combined method of transcript profiling and computational analysis. Computational analysis depends on the prediction of signal peptides, which is viewed as a hallmark of classically secreted proteins. According to the famous signal hypothesis [20], the majority of secreted proteins have an N-terminal signal peptide sequence that helps proteins to enter the endoplasmic reticulum (ER) lumen via the sec-dependent protein translocation complex. Welsh et al [22] used a combined method of controlled vocabulary terms and sequence-based algorithms to predict genes encoding secreted proteins from 12,500 sequences on oligonucleotide microarrays in common human carcinomas. They successfully identified 2,300 genes, of which 74 were over-expressed in one or more carcinomas. Another similar study found a total of 133 statistically significant secretome genes correlating to breast cancer progression [23].

These genome-based methods can provide a comprehensive list of potentially secreted proteins quickly. However, there are two major inherent problems that restrain the broad use of these approaches. First, this approach relies on prediction of signal peptides or cell retention signals, thus making some genuine secreted proteins lacking signal peptide or presenting cell retention signals unpredictable. About 50% of secreted proteins can be predicted by signal peptides or other specific cell retention signals [24]. Second, secreted proteins are frequently regulated at the post-transcriptional level. Accordingly, the real level of expression of secreted proteins does not always correlate with mRNA expression [25,26]. The inconsistent expression pattern between mRNA and protein will inevitably hamper the clinical application of biomarkers from these genome-based prediction methods.

### Proteomic approaches

Nowadays, proteomic technologies are the mainstay of cancer secretome studies. With the massive progress in mass spectrometry (MS), bioinformatics and analytical techniques, proteomic approaches greatly promote the cancer secretome analysis and biomarker discovery. Currently, there are roughly three major proteomic technologies in secretome researches: gel-based methods, gel-free MS-based methods and surface-enhanced laser desorption/ionization time-of-flight mass spectrometric (SELDI-TOF-MS).

#### Gel-based proteomic technologies

Two-dimensional gel electrophoresis (2-DE) coupling MS is the most classic and well-established proteomic approach. This method allows the separation of complex mixtures of intact proteins at high resolution. These protein mixtures are first separated according to their charge in the first dimension by isoelectric focusing (IEF) and size in the second dimension by SDS-PAGE, and then analyzed by peptide mass fingerprinting using MS or MS/MS after in-gel trypsin digestion. It has been widely used in secretome studies of cancers, such as malignant glioma [26], lung cancer [27-29], hepatocellular carcinoma [30], fibrosarcoma [31], breast cancer [32] and oral squamous cell carcinoma [33]. Using 2-DE coupled to matrix-assisted laser desorption/ionization time-of-flight mass spectrometry (MALDI-TOF-MS), Huang [27] et al. identified 14 human proteins from the conditioned media of a non-small cell lung cancer cell line A549. With the same technique, Lou et al [28] identified 47 proteins from the conditioned media of M-BE, an SV40T-transformed human bronchial epithelial cell line with the phenotypic features of early tumorigenesis at high passage.

Although 2-DE currently remains the most efficient method for separation of complex protein mixtures, it is clear that this technique has several disadvantages, including poor reproducibility between gels, low sensitivity in the detection of proteins in low concentrations and hydrophobic membrane proteins, limited sample capacity and low linear range of visualization procedures [34]. In addition, the technique is time-consuming, labor-intensive and has a low efficiency in protein detection due to limited amenability to automation.

To circumvent some of these inherent problems of the standard 2-DE procedure, a modified method, differential in-gel electrophoresis (DIGE) has been developed by GE Healthcare [35]. This technology utilizes three spectrally distinct, charge and mass-matched fluorescent dyes (Cy2, Cy3 or Cy5), which can primarily combine covalently with lysine. Protein samples are differently labeled by these fluorescent dyes before electrophoresis, and then mixed and separated on one single gel. By enabling two protein samples to run on the same gel, DIGE significantly reduces the experimental variations and ensures that the biological difference becomes the predominant contribution to the total variance. Fluorescent labeling also enhances the linear dynamic range and detection sensitivity in DIGE [36]. Volmer et al [21] performed a differential secretome analysis between the smad-4 deficient and smad-4 re-expressing SW480 human colon carcinoma cells by both DIGE and traditional 2-DE technologies. After systematically comparing the protein patterns and the performance of the two methods, they convincingly demonstrated that DIGE was more reliable and powerful than traditional 2-DE. Despite DIGE being envisaged as a more powerful technique than conventional 2-DE for proteomic studies, it still has a number of shortcomings. First, the technique is not applicable to those proteins without lysine (when labeling with the minimal dyes) or cysteine (when labeling with the saturation dyes). Second, DIGE still suffers from some problems inherent to 2-DE, such as low throughput and difficulties in the identification of proteins with extreme isoelectric points or molecular weight. This fact has necessitated the development of alternative proteomic strategies to achieve information not accessible through 2D gel separation.

#### Gel-free MS-based technologies

To overcome the inherent drawbacks of gel-based approaches, great efforts have been made recently on gel-free MS-based or shotgun proteomics. In these newly emerging approaches, instead of depending on gels to separate and analyze proteins, complex mixtures of proteins are first digested into peptides or peptide fragments, then separated by one or several steps of capillary chromatography, and finally analyzed by MS/MS. Multidimensional protein identification technology (MudPIT), which was introduced and termed by Yates and colleague [37], is one of the most typical approaches in gel-free technology. In MudPIT, strong cation exchange (SCX) and reversed-phase (RP) liquid chromatography (LC) are coupled with automated MS/MS to adequately separate peptides from the peptide mixtures by charge and subsequent hydrophobicity. Thousands of peptides were quickly identified for a given sample by using the SEQUEST algorithm to analyze the MS/MS data. Because of its high-resolution separation of peptides and the significantly enhanced protein coverage, MudPIT is powerful in the analysis of membrane proteins or low-abundance proteins/peptides which are undetectable in gel-based approaches [38,39]. Thus, MudPIT has now become the popular technology in the investigation of the cancer secretome [40-43]. However, essentially, MudPIT is not a quantitative proteomic approach. Hence, it is not regarded as optimal for differential proteome analysis [44]. Bioinformatics algorithms were recently developed to overcome this limitation by showing its promising application in differential proteomic analysis. These methods were simply based on mass spectral signal intensity or peptide hits, and thus were categorized as LC-MS/MS based non-labeled quantitative proteomic quantification [45,46]. However, much work needs to be done if these algorithms are to be broadly accepted in the future.

The major progress in proteome/secretome study is the technology of quantitative proteomics which introduced isotopes or other molecular labeling methods in proteomic analysis [47-49]. In these methods, proteins or peptides from different samples are first labeled with different stable isotopes or chemicals, then mixed, separated and identified by single dimension or multidimensional LC coupling MS/MS. By having the same chemical properties, a peptide in a mixed pool detected by MS appears as peak pairs (peptides existing distinctly in one sample are detected as single peaks). The measurement of either the MS peak intensities or areas can infer relative abundance between protein samples [48]. One of the most extensively applied approaches in stable isotope labeling technologies is isotope-coded affinity tag (ICAT), which was introduced by Gygi and colleagues in 1999 [50]. The ICAT reagent consists of three parts: a reactive group specific for free thiol functionality of cysteine residues, a linker and a biotin tag that makes possible affinity chromatography purification using immobilized avidin. By labeling with isotopically light- or heavy-ICAT reagent, the amount of two protein samples can be compared with the MS data. Being specific for cysteine residues, ICAT reagents can neglect the sample complexity and allow detection of low-abundance peptides [51]. Martin and colleagues [52] comprehensively analyzed androgen-regulated secreted proteins from neoplastic prostate tissue by the ICAT approach. They successfully identified 52 androgenic hormone regulated proteins including PSA, neuropilin-1, amyloid-like protein 2, and prostate differentiation factor. Recently, a second-generation ICAT reagent called cleavable isotope-coded affinity tag (cICAT) has been developed. Differing from the original reagents, the cICAT reagent uses an acid-cleavable linker and^13^C or ^12^C isotopes [53,54]. This approach shows enormous potential for quantitative proteomic analysis, and a cICAT-based secretome study in human glioma cells found 47 proteins with significant expression changes in response to p53 expression [26]. However, this technique is not very efficient for proteins with few or no cysteines [55].

Stable isotope labeling by amino acids in cell culture (SILAC) is another common stable isotope labeling technique. In SILAC, stable isotope-labeled essential amino acids are added to amino acid deficient cell culture media, and then are absorbed and secreted by cells in the synthesis of proteins in vitro. Thus the proteome from different cell cultures can be compared as being grown in media with carbon-isotopically modified amino acids. A differential SILAC secretome study between pancreatic cancer cells and non-neoplastic pancreatic ductal cells identified 145 differentially secreted proteins (> 1.5-fold change), including several common biomarkers of pancreatic cancer and novel proteins that have not been reported previously [25]. Nearly all peptides can be isotopically labeled by SILAC, hence significantly improving the sequence coverage of proteins. SILAC might be the best method for secretome study in vitro at present; however, this approach is impractical for clinical protein samples in vivo.

Isobaric tag for relative and absolute quantization (iTRAQ) is a recently developed isotope labeling approach that is increasingly accepted in secretome analysis [56]. This new method can label nearly all peptides in a digested mixture from either cell lines or clinical samples. It also allows for multiplexing the analysis of up to four samples in a single experiment by employing a 4-plex set of amine reactive isobaric tags, and the mass spectra of peptides generated are relatively easy to interpret [57]. iTRAQ has been applied to investigate the secretome differences between Pseudoalteromonas tunicata wild-type (wt) and the white mutant (wmpD-), and identified 182 proteins with > 95% confidence [58]. Nevertheless, to our knowledge, applications of this new technique are not as yet reported in cancer secretome studies.

#### SELDI-TOF-MS

SELDI-TOF-MS is an exciting approach in cancer proteomics, particularly plasma proteomics [59-61]. The paradigm of this method is the protein chip arrays, which have specific chromatographic features. After an on-surface chromatographic protein separation, the chip-immobilized proteins are co-crystallised with a matrix and the MS spectral profiles are captured by an analyzer. By analyzing these spectral profiles, a cancer-specific finger-print can be obtained. SELDI-TOF-MS has several advantages, including relatively high tolerance for salts and other impurities, improved sensitivity for lower-abundance proteins, no requirement for off-line protein isolation and compatibility with automation [62]. However, its major disadvantage lies in the fact that it is difficult to identify the potential biomarkers from the differential spectral profiles, and thus was suspected by some investigators [63,64]. Fortunately, recent studies seemed to overcome this obstacle [65,66]. Moscova et al [66] successfully separated five PI3K-regulated secreted proteins (CXCL1, IL-8, and variant forms) in ovarian cancer cells from SELDI-TOF-MS spectral profiles by proteomic and immunologic methods. These molecules might be used either as diagnostic markers or as targets for the pathway-specific molecular therapies. The high-throughput nature and simplicity in its experimental procedures hold out SELDI-TOF-MS to be a promising technology for future secretome analysis and biomarker discovery.

## Applications of cancer secretome analysis

### Identification of cancer biomarkers

The major application of cancer secretome analysis is to search for cancer biomarkers. As mentioned above, the cancer secretome contains a treasure trove of novel biomarkers, which make cancer diagnosis using secretome markers attractive. Recently, investigation of secretomes from a variety of cancers has led to the identification of a number of potential cancer biomarkers (Table 1). It is known that renal cell carcinoma (RCC) is the sixth leading cause of cancer-related deaths, and metastasis is found in 15%–25% of RCC patients at the time of diagnosis. To date, no validated RCC marker is available to detect asymptomatic RCC [67]. Aiming to explore novel circulating RCC markers, Sarkissian et al [68] analyzed the secretome of CAL 54, a human RCC cell line and identified pro-matrix metalloproteinase-7 (pro-MMP-7) as a candidate serum marker. By employing a homogeneous, fluorescent, dual-monoclonal immunoassay, the concentrations of pro-MMP-7 in serum samples were examined. The concentrations of pro-MMP-7 were found to be increased in serum of RCC patients compared with healthy controls, and serum pro-MMP-7 had a sensitivity of 93% (95% CI 78–99%) at a specificity of 75% (59–87%) for RCC, indicating pro-MMP-7 might be a promising RCC marker. Biomarkers for nasopharyngeal carcinoma are also urgently needed. Wu et al [69] combined SDS-PAGE with MALDI-TOF-MS to systematically investigate the nasopharyngeal carcinoma secretome. From the cultured media of nasopharyngeal carcinoma cell lines, they identified 23 proteins and found that 3 metastasis-related proteins, fibronectin, Mac-2 binding protein (Mac-2 BP), and plasminogen activator inhibitor 1 (PAI-1), were overexpressed in nasopharyngeal carcinoma tissues. ELISA-based detection further indicated that the serum levels of these proteins were significantly elevated in nasopharyngeal carcinoma patients than in healthy controls, highlighting their potential for nasopharyngeal carcinoma detection.

**Table 1:** Candidate biomarkers for human cancers discovered by cancer secretome analysis

Cancer	Screening methods	Verification methods	Candidate biomarkers	References
Lung	SDS-PAGE/nano-ESI-MS/MS	ELISA	CD98, fascin, 14-3-3 η, polymeric immunoglobulin receptor/secretory component	[73]
	2-DE/MALDI-TOF/TOF-MS	Western blot/ELISA/IHC	Cathepsin D	[28]
	2-DE/MALDI-TOF-MS	RT-PCR/western blot/ELISA/IHC	Dihydrodiol dehydrogenase	[27]
	SDS-PAGE/MALDI-TOF-MS	ELISA	L-lactate dehydrogenase B	[90]
	2-DE/MALDI-TOF-MS	RT-PCR/enzyme activity detection	Mn-SOD	[29]
Liver	LC-MS/MS	Western blot	Apolipoprotein E, DJ-1, apolipoprotein H, galectin-3, cathepsin L, cyclophilin A, cystatin C	[41]
Pancreatic	NuPAGE/LC-MS/MS/SILAC	Western blot/IHC	CD9, perlecan, SDF4, apolipoprotein E, fibronectin receptor, Mac-2 binding protein, cathepsin D, cathepsin B, MCP-1, L1CAM	[25]
	LC-MS/MS	RT-PCR/western blot/IHC	CSPG2/versican, Mac25/angiomodulin	[43]
Bladder	SDS-PAGE/MALDI-TOF-MS	Western blot	Pro-u-plasminogen activator	[91]
	LC-MS/MS		CXCL1	[92]
Nasopharyngeal	SDS-PAGE/MALDI-TOF-MS	Western blot/ELISA/IHC	Fibronectin, Mac-2 binding protein, plasminogen activator inhibitor 1	[69]
Prostate	LC-MS/MS	Western blot/ELISA	Mac-2 binding protein	[40]
	Oligonucleotide microarray/genome-based computational prediction	RT-PCR/ELISA/IHC	Macrophage inhibitory cytokine 1	[22]
	LC-MS/MS	ELISA	follistatin, chemokine (C-X-C motif) ligand 16, pentraxin 3, spondin 2	[93]
Melanoma	NuPAGE/LC-Q-TOF-MS/MS	Western blot	Cathepsin D, gp100	[79]
Breast	LC-MS/MS	Western blot	Galectin-3-binding protein, alpha-1-antichymotrypsin	[94]
	LC-MS/MS	ELISA	Elafin	[95]
Colorectal	SDS-PAGE/MALDI-TOF-MS	Q-PCR/Western blot/IHC/ELISA	Collapsing response mediator protein-2	[72]
	2-DE/DIGE/MALDI-TOF-MS	Northern blot/western blot	Cathepsin D, stratifin, calumenin	[21]
Renal	2-DE/MALDI-TOF-MS/immunoblotting	Western blot/homogeneous fluorescent immunoassay	Pro-MMP-7	[68]
Oral	SDS-PAGE/MALDI-TOF-MS	Western blot/IHC/ELISA	Mac-2 binding protein	[70]
Fibrosarcoma	Capillary ultrafiltration probe/2-DE/MALDI-TOF-MS		Cyclophilin A, S100A4, profiling-1, thymosin beta 4, thymosin beta 10, fetuin-A, alpha-1 antitrypsin 1–6, contrapsin, apolipoprotein A-1, apolipoprotein C-1	[31]
Ovarian	SELDI-TOF MS	Immunodepletion	CXC chemokine ligand 1, intact and truncated interleukin 8	[66]
	HPLC fractionation/LC-MS/MS	Immunoblot/immunofluorescence	14-3-3 zeta	[96]

As shown in table 1, several putative biomarkers unraveled in cancer secretomes are commonly shared among different cancers, such as Mac-2 binding protein [25,40,43,69,70], cathepsin D [21,25,28,71] and apolipoprotein E [25,41]. To identify unique markers for colorectal cancer, the secretomes of 21 cancer cell lines derived from 12 cancer types (colon cancer, leukemia, bladder cancer, lung cancer, NPC, hepatocellular carcinoma, cervical carcinoma, epidermoid carcinoma, ovary adenocarcinoma, uterus carcinoma, pancreatic carcinoma and breast cancer) were compared. Based on its selective secretion in the colorectal cell line secretome but not in the other tested cell lines, collapsin response mediator protein-2 (CRMP-2) was selected for further evaluation. Q-PCR and immunohistochemical (IHC) staining confirmed the high expression of CRMP-2 mRNA and protein in colorectal tissues. Fluorimetric competitive ELISA was performed to examine the levels of CRMP-2 and CEA in plasma samples from colorectal patients and healthy controls. The sensitivities of plasma CRMP-2 and CEA were found to be 60.5% and 42.9%, respectively, indicating that CRMP-2 could be a colorectal marker superior to CEA. Additionally, the combination of CEA and CRMP-2 for CRC screening showed a higher capacity than either marker alone by enhancing the sensitivity and specificity from 42.9 to 76.8% and 86.6 to 95.1%, respectively [72].

There is a growing consensus that no single cancer biomarker is sensitive and specific enough to meet stringent diagnostic criteria given the substantial heterogeneity among cancers. A feasible strategy to circumvent the drawbacks of individual markers is to measure a combination of proteomic biomarkers. To get panels of serum biomarkers for lung cancer detection, Xiao et al [73] compared the secretome of lung cancer primary cell or organ cultures with that of the adjacent normal bronchus using one-dimensional PAGE and nano-ESI MS/MS. They totally identified 299 proteins, in which 13 interesting proteins were selected for investigation in 628 plasma samples with ELISA. Eleven of these 13 proteins were detected in the plasma samples, only without nm23-H1 and hnRNP A2/B1 possibly because they were below the present sensitivity threshold. After using Tclass classification system to analyze all possible feature combinations of these 11 proteins, they found that a combination of four proteins, CD98, fascin, polymeric immunoglobulin receptor/secretory component and 14-3-3 η had a higher sensitivity and specificity than any single marker. Thus, investigating cancer secretome provides a useful tool to establish cancer marker profiles for high-quality cancer detection.

Taken together, these studies demonstrate that secretome analysis is a feasible and efficient method to find, identify, and characterize clinical relevant biomarkers.

### Investigation of the mechanisms on carcinogenesis and gene functions

In addition to the identification of candidate biomarkers, cancer secretome analysis can provide new insights into the molecular mechanisms of carcinogenesis. Extracellular events such as cell-to-cell interactions and cell-to-extracellular matrix interactions are crucial during carcinogenesis. To characterize extracellular events associated with breast cancer progression, secreted protein-encoded gene expression profiles were investigated in a cell line model of human proliferative breast disease (PBD). Differentially expressed genes from microarray data were searched for genes encoding secreted proteins in three public databases. The analysis displayed two clusters of secretome genes with expression changes correlating with proliferative potential, implicating a role in breast cancer progression [23]. In a recent secretome study [74], two UV-induced fibrosarcoma cell lines (UV-2237 progressive cells and UV-2240 regressive cells) were used as models to investigate aspects that affect tumor formation. In addition to analysis of differential proteome expression in these two cell lines, in vivo secretome from samples collected from tissue chamber fluids was characterized and quantified via an isotope-coded protein label (ICPL) in conjunction with high-throughput NanoLC-LTQ MS analysis. Three differential proteins in secretome including myeloperoxidase, alpha-2-macroglobulin, and a vitamin D-binding protein, together with 25 differential proteins in the proteome between these two cells were identified, partially revealing a possible mechanism underlying the succession and attenuation of cancers.

Differential cancer secretome analysis can also advance our understanding on the functions of interesting genes. It is known that tumor-suppressive p21 is a negative regulator of cell cycle progression; however, several studies have shown that p21 expression in tumor cells mediates an anti-apoptotic and mitogenic paracrine effect [75,76]. In order to clarify such paradoxical phenomena, Currid et al [65] have characterized secretomes of HT-1080 human fibrosarcoma cells displaying inducible p21 expression by SELDI-MS technology. Three putative p21-regulated factors (cystatin C, pro-platelet basic protein, beta-2-microglobulin) were identified and validated, which have been shown previously to have growth-regulating effects and might contribute to the observed mitogenic and anti-apoptotic paracrine activity of p21-expressing cells. To study the role of p53, a major tumor suppressor, in carcinogenesis through its manipulation of the tumor microenvironment, Khwaja et al [26] compared secretomes of p53-null tumor cells in the presence or absence of reconstituted wt-p53 expression. Using 2-DE in conjunction with cICAT, they found 50 p53-controlled secreted proteins. These proteins have known roles in cancer-associated processes such as immune response, angiogenesis, cell survival, and extracellular matrix (ECM) interaction. Interestingly, most of these proteins were found secreted through receptor-mediated nonclassical secretory mechanisms, indicating a role of p53 in the regulation of the nonclassical secretory pathway.

## Challenges and perspectives

### Preparations for in vitro cancer secretome samples

**Figure 1:**
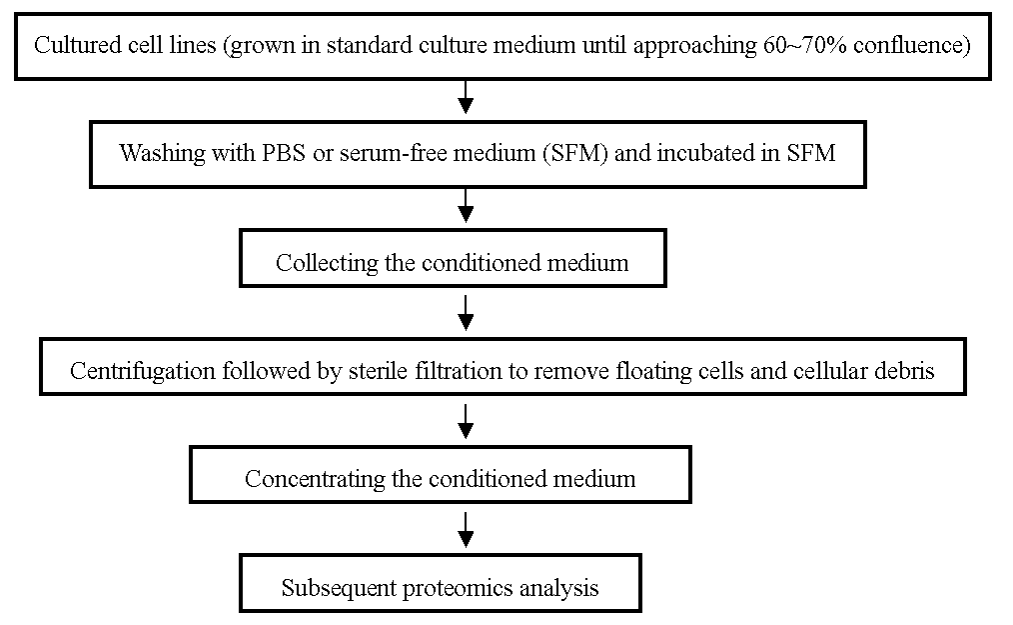
Secretome preparation from the conditioned media of in vitro cells culture.

To gain reliable insights into the cancer secretome, it is first necessary to prepare samples for analysis which are as pure as possible. Secreted proteins in vivo occur in body fluids, thus the direct analysis for them is hindered by the high complexity. It is generally accepted that proteins secreted by tumor cells in vitro may, to some extent, reflect the proteins released by tumors in vivo. Therefore, the routine method used to date is to obtain secreted proteins from the media of in vitro cancer cell culture(Figure 1).

Although cells are commonly cultivated in serum-supplemented media, serum-free media (SFM) are needed to guarantee the successful analysis of the cancer secretome in vitro. The reason lies in the fact that the highly abundant serum proteins such as albumin may mask and dilute the secretome, whereas cell growth is much slower in SFM, and these cells tend to autolyse and liberate cytosolic proteins. Mbeunkui et al [42] performed a comprehensive study of the secretome of three metastatic cancer cell lines in vitro. To obtain minimal cytosolic protein contamination, they optimized the incubation time and the cell confluence. Two cytosolic proteins beta-actin and beta-tubulin were applied to monitor cell lysis. Comparing the LC-MS/MS analysis of the secretome under different culture conditions in SFM, they found that the level of these two cytosolic proteins increased noticeably in the culture media after 30 hours incubation or when the cell confluence was above 70%. Finally, an incubation time of 24 hours and 60–70% cell confluence were considered as optimal cell incubation conditions. Mauri et al [43] also investigated several different preparations of secretome from cancer cell lines. In their study, the 18 hours time point was the longest incubation time generating a good signal in MudPIT analysis without obvious signs of cell lysis. These results tell us that the optimal conditions vary according to specific studies. Morphological and dye exclusion assay evaluation, as well as the detection of some cytosolic proteins can help us to determine the optimal conditions.

In consideration of the significant masking effects of bovine serum albumin (BSA) and other serum constituents, washing the cells thoroughly to reduce serum contaminations before incubation in SFM is a necessary step, whereas stringent washes can damage or kill the cells and lead to the nonspecific liberation of cytoplasmic proteins. Thus, how to keep a balance between serum contaminations removal by washing and cell survival is the key. Pellitteri-Hahn et al [77] used rat endothelial cells as a model to compare three different rinsing methods: in the first group, no rinsing treatment was given; the second group received a moderate rinsing treatment; the last group, in a stringent rinsing treatment, was rinsed twice with 10 mL of Dubelcco's phosphate buffered saline with calcium and magnesium (DPBS) and once with 10 mL of SFM. They demonstrated that the percentage of contaminant BSA was much lower in the stringently rinsed cells (average 13.2%) compared with either the moderate or no-wash treatment (average 35.2 and 45.2%, respectively). More importantly, the reduction of BSA in the stringent wash group increased the protein identification significantly without apparently interrupting cell growth or viability. Therefore, it is important to adequately wash the cells, and the stringent method described in this study proved to be a desirable one, keeping the balance between serum protein reduction and cell survival.

There is no doubt that optimizing the cell culture conditions and employing an appropriate washing technology can significantly reduce serum or cytosolic protein contamination. Nevertheless, some serum constituents are still present in culture media even after thorough rinsing treatment, and even under optimum culture conditions, cell cultivation in vitro is unavoidably accompanied by cell death and subsequent release of cytosolic proteins. Because the concentration of secreted proteins is always very low, the contamination by non-secreted proteins may easily mask the proteins of interest. Consequently, how to discriminate genuine secreted proteins from non-secreted proteins is a major question that remains to be answered. Zwickl et al [30] have established a metabolic labeling-based technology which allows for the sensitive and selective detection of authentic secreted proteins. They demonstrated the applicability of this method through a study on the secretome of the hepatocellular carcinoma-derived cell line HepG2 and human liver slices. In their study, HepG2 cells were incubated in serum-free, methionine- and cysteine-free RPMI-1640 in the presence of [35S]-labelled methionine and cysteine, then the cell supernatant was filtered, precipitated, and subjected to two-dimensional gel electrophoresis. Finally, the gel was stained with RuBPS and proteins detected by fluorescence analysis and autoradiography. While fluorescence analysis detects all proteins which may contain a large number of cytosolic or serum proteins, autoradiography detects only those proteins synthesized by living cells during the metabolic labeling period. Indeed, all identified 16 protein spots, which showed positive radiolabels, were found to be authentic secreted proteins. Therefore, the application of this novel approach can improve cancer secretome analysis by specifically detecting and identifying genuine secreted proteins.

Secreted proteins present in the culture media are usually in low concentrations, which can go down to the ng/mL range, as in the case of some cytokines. Thus, proteins secreted in the culture media should be concentrated before subsequent proteomics analysis. Various methods have been used to concentrate the proteins; nonetheless, these methods are not all well suited for the secretome analysis. For example, precipitation with acetone can not concentrate large volumes of culture medium because a minimum five-fold volume excess of acetone should be used, and dye precipitation selects against an important class of secreted proteins – the proglycoproteins [78]. Among these methods, ultrafiltration is most often used in the concentration of the secretome [41,79,80]. It is proved to be an efficient technology despite the leakage of low molecular weight proteins. Mireille et al [81] described an improved technology for secretome concentration, which is based on carrier-assisted TCA precipitation. In this study, 5 protein concentration technologies were evaluated for the performance and compatibility with 2-DE, and carrier-assisted TCA precipitation was clearly superior to the others. This technology did not distort the protein patterns, and enabled the identification of secreted proteins at concentrations close to 1 ng/mL such as TNF and IL-12. However, this technology still missed some proteins; in fact, cytokines such as IL-1 and IL-6 have not been detected.

### In vivo cancer secretome studies

Currently, most studies on the cancer secretome involve collecting secreted proteins from supernatants of cancer cell lines cultivated in vitro and then analyzing their properties in vivo. Nevertheless, the in vitro cell culture systems are far from physiological situations. Then, the question is whether the in vitro cell culture systems are able to completely replicate the in vivo conditions, or whether the data from in vivo secretome can match well with that achieved in vitro. Considering the great challenges for obtaining pure secretome, to date, only a minority of studies have investigated cancer secretome under in vivo situations. Varnum et al [82] characterized the protein pattern of the nipple aspirate fluid (NAF), that contains proteins directly secreted by the ductal and lobular epithelium, in women with breast cancer. Using gel-free proteomic technologies, they identified a total of 64 proteins. Among these proteins, 15 proteins, including cathepsin D and osteopontin, have been previously reported to be potential markers for breast cancer in serum or tumor tissues. Celis et al [83] employed 2-DE and MALDI-TOF-MS to analyze the tumor interstitial fluid (TIF), which was collected from small pieces of freshly dissected invasive breast carcinomas. TIF perfuses the breast tumor microenvironment, and consists of more than one thousand proteins. From TIF, they identified 267 primary translation products, involved in cell proliferation, invasion, angiogenesis, metastasis and inflammation. A novel technology for investigating in vivo cancer secretome was developed by Huang and colleagues [31]. They collected in vivo secretome directly by implanting capillary ultrafiltration (CUF) probes into tumor masses of a live mouse at the progressive and regressive stages. With MS proteomics, ten secreted proteins were identified. Among them, five proteins, including cyclophilin-A, S100A4, profilin-1, thymosin beta 4 and 10, which previously correlated to tumor progression, were identified at the progressive stage. The remaining five secreted proteins (fetuin-A, alpha-1-antitrypsin 1–6, and contrapsin) were identified at the regressive stage. The approach using CUF probes to capture in vivo secreted proteins from a tumor mass sheds light on in vivo secretome examinations and cancer biomarker discovery.

### Validation for biomarkers discovered from cancer secretome

For achieving reliable and clinically worthwhile biomarkers, the interesting protein markers discovered from the cancer secretome need to be further validated. To some extent, validation is more arduous than discovery [84], and there have been concerns regarding the biomarker validation process. First, immunoassays based on specific antigen and antibody reaction are routinely employed for biomarker verification, whereas, the specific antibodies with the required affinity and specificity for the targets are not usually available. To overcome the reagent limitations, methods that do not demand antibodies continue to be explored. Undoubtedly quantitative MS analysis using multiple reaction monitoring (MRM) presents a compelling alternative. This approach employs synthetic isotope-labeled peptide as internal standard, allowing very accurate measurements of target proteins. Multiplexing and high-throughput are major advantages of this approach, which enable characterization of a number of candidate proteins simultaneously. Although quantitative LC-MRM MS has been demonstrated to be a powerful tool for biomarker validation, its sensitivity compared to existing immunoassays is still a matter of concern [85-87]. Second, adequate and reasonable clinical tissue or plasma specimens (patient group and matched controls) are crucial to biomarker validation. However, the availability of high-quality specimens with well-matched controls is limited [88]. Finally, the proteomics platform currently used is far from comprehensive and lacking high-throughput – hence it is unable to handle a large number of samples during the biomarker validation process [89].

## Conclusion

Analysis and characterization of a cancer secretome is a critical step towards the biomarker discovery process, which represents a challenge for current technologies. Though genome-based approaches are convenient and comprehensive, the accuracy for predicting secreted proteins is always far from satisfactory owing to the inherent drawbacks. Furthermore, there is always a discrepancy between the expression levels of mRNA and the corresponding secreted proteins. For allowing direct analysis for secreted proteins, proteomic methods are considered as a more powerful means to investigate the cancer secretome. While classic gel-based proteomic technologies have produced significant contributions to biomarker discovery, the emergence of gel-free MS-based proteomic approaches, such as MudPIT and SELDI-TOF-MS, greatly facilitates the secretome analysis with increased sensitivity and automation. Proteomic approaches currently used are not as rapid and high-throughput as genomic profiling with microarrays – hence improving proteomic methods towards higher comprehensiveness, throughput, reproducibility and accuracy is of vital importance. Considering genomic-based and proteomic approaches provide closely related but distinct information about the cancer secretome, they can be combined as complementary methods. Searching for biomarkers from cancer secretome analysis also challenges bioinformatics, which needs to cope with the vast amounts of data from MS. To gain more reliable insights into the cancer secretome and develop valuable cancer biomarkers, the optimization of sample preparation procedure should be fully established, and more efforts should be focused on in vivo secretome research and biomarker validation. Overall, investigating the cancer secretome opens up new avenues in the search for clinically worthwhile biomarkers. With the rapid development of new strategies and technologies, this newly emerging field will reveal more valuable information on cancer diagnosis, monitoring and therapy.

## Competing interests

The authors declare that they have no competing interests.

## Authors' contributions

HX wrote the manuscript. BJL edited the manuscript. MDL organized and revised the manuscript. All authors read and approved the final manuscript.
